# Sublamine as a Conjunctival Disinfectant

**Published:** 1904-01

**Authors:** Josef Imre

**Affiliations:** Hódmezövásárhely, Austria, Physician-in-chief to the Elizabeth Ophthalmological Hospital


### ABSTRACTS.

### Sublamine as a Conjunctival Disinfectant.

By DR. JOSEF IMRE, at Hódmezövásárhely, Austria,
Physician-in-chief to the Elizabeth Ophthalmological Hospital.

[From *Die Heilkunde*, September, 1903.]

IN conjunctivitis attended with copious secretion we need an antiseptic which is as energetic as corrosive chloride and as harmless as boric acid. Such a one is sublamine, which I have used in the hospital for the past year. Sublamine, the ethylene-diamine-sulphate of mercury, has already proved superior to bichloride for hand disinfection. It does not irritate the skin, may be employed as strong as is desired, has a deeper action than sublimate and possesses other advantages. I use it for the treatment of conjunctivitis, for preparing the field of operation on the eye, cleansing the lachrymal sac, and for similar purposes.

Sublamine is most valuable in the various forms of suppurative conjunctivitis. In these cases everything depends upon irrigation and the frequent removal of accumulated secretions. The earlier this is begun the better of course it is; but even when begun only late it is essential to prevent the stagnation of the noxious substances in the sac. In acute cases with abundant suppuration, as in gororrheal conjunctivitis, sublamine, 1-1000 answers admirably, being readily borne. Instillations are made from a large eye glass onto the everted lid; where this cannot be done the lids are opened with the fingers or spoons. Treatment is kept up day and night, 1½ to 3 ounces being used every half hour and occasionally oftener to obviate pus stagnation; but experience has taught me that too frequent irrigation no matter with what causes the formation of pseudomembranes.

In numerous cases of blenorrhea neonatorum sublamine did us the greatest service. Treatment in the newborn is comparatively easy, especially in the less virulent forms and those that come under treatment early. But the eyes of children which cannot be seen daily are in great danger. In these cases the attendant is instructed thoroughly how the sublamine irrigations are to be performed and for the first few days the patient is seen every day. When I am convinced that treatment is carefully carried out and the cornea is unhurt, the patient comes only twice a week. Of course in severe cases the pencillings with silver nitrate cannot be omitted.

In our hospital each trachoma patient gets, in addition to abundant saline irrigations twice daily, a bottle of 1-2000 sublamine together with cotton and a pus basin, so that he can wash his eyes as often as is necessary. We formerly used boric acid; but since we give sublamine and the assistant physician or the nurse cleanses the conjunctiva with it before morning and afternoon rounds, even freely secreting, verrucous, plicated and thickened conjunctiva clear up and are cured decidedly faster. The conjunctiva does not stand effective concentrations of corrosive sublimate, but patients never complain of sublamine. In other severe infections, conjunctivitis and generally in all cases of fresh catarrh, sublamine compresses and irrigations are more effective than boric acid irrigations and lead or zinc. The secretion disappears more rapidly and the conjunctiva thins quicker. Painting with a weak silver nitrate solution, or better with argentamine, must not be omitted; otherwise the later stages of the disease, when the violent irritative symptoms have disappeared, may be much protracted.

